# Editorial: Diet and nutrition for non-communicable diseases in low and middle-income countries

**DOI:** 10.3389/fnut.2023.1179640

**Published:** 2023-03-28

**Authors:** Rahnuma Ahmad, Farhana Akter, Mainul Haque

**Affiliations:** ^1^Department of Physiology, Medical College for Women and Hospital, Dhaka, Bangladesh; ^2^Department of Endocrinology, Chittagong Medical College Hospital, Chattogram, Bangladesh; ^3^Unit of Pharmacology, Faculty of Medicine and Defence Health, Universiti Pertahanan Nasional Malaysia (National Defence University of Malaysia), Kuala Lumpur, Malaysia

**Keywords:** low-to-middle-income countries, malnutrition, diet, poor choices, urbanization, knowledge lack, prevention, supplementation

The indicators of nutritional status, which include underweight, overweight, short stature, and obesity, are changing in low-to-middle-income countries (LMICs) (1). These indicators need to be researched with recent data and compared with past data to observe the changing scenario for nutritional status in these countries. Moreover, it is important to understand the government policies on food that are in place to overcome the burden of malnutrition and the extent of their application in the public health field. Unhealthy food consumption, including that of sugar, fast food, fat, and oil, in LMICs needs to be assessed.

Both undernutrition and overnutrition appear to be a growing burden on LMICs (2). Globally, about one-third of the population is affected by malnutrition, with approximately one billion individuals suffering from undernutrition due to inadequate protein, calorie, and micronutrient intake (3). The LMICs are facing the hurdles of the poor growth of children, deficiency of micronutrients, and adults with high BMI (4).

There has also been a rise in non-communicable disease (NCD) in LMICs owing to their populations suffering undernutrition at the early stages of life (in early childhood and mothers suffering undernutrition before and during pregnancy) and then becoming overweight during adulthood (5). An increased risk of growth stunting, NCD, and obesity in later life is suffered by children whose mothers were undernourished during pregnancy (6). Rapid weight gain in children may be related to the raised risk of cardiometabolic diseases and obesity later in life (7). Oumer et al. performed research on the dietary pattern among pregnant women in Eastern Ethiopia to understand the dietary pattern there and the possible factors influencing the dietary intake, since it is important to create a nutritional balance during pregnancy to prevent adverse pregnancy outcomes and the poor growth and development of children in the future.

Obesity among children is a growing concern, and children in LMICs are exposed to an unhealthy diet high in sugar and fat (8). Children suffering from obesity face the danger of developing chronic diseases in later life. Advertisements that encourage unhealthy food consumption are bombarding children, and thus there is a need for regulatory policies to keep the level of advertisements in check. Al-Jawaldeh and Jabbour analyzed the policies in place to regulate the marketing of food that targets children in the EMR (Eastern Mediterranean Region) to examine the extent of the implementation of the WHO-recommended food marketing policies framework that targets children. Obese and overweight children and adolescents become more susceptible to hypertension. Thus, the BMI of obese and overweight adolescents must be checked and kept within the normal range to combat the development of this non-communicable disease (9). However, measuring BMI is quite a tedious task, since there are multiple cut-off points in the case of children and adolescents. Recently, the tri-ponderal mass index has been reported to be more straightforward, with only four cut-off points, and more effective for the screening of body fat among those in the 7–18 age group (10, 11). Hu et al. performed a study comparing BMI and tri-ponderal mass index results for discrimination of hypertension among adolescents. They concluded that the tri-ponderal mass index has a better ability in hypertension prediction when compared to the use of BMI (Hu et al.).

The deficiency of common micronutrients is of public health significance in LMICs (12). Important micronutrients that may be deficient include iodine, iron, vitamin A, vitamin D, folate, and zinc. Such deficiencies may be the outcome of insufficient health care, poor quality of diet, lack of awareness, and poor sanitation (13). A deficiency of such micronutrients may lead to poor pregnancy outcomes, poor growth and development in children, and other health disorders, including goiter, poor vision, skin lesions, and even mental conditions such as depression (14). Zhang et al. delved into research to find a relationship between vitamin A and beta-carotene intake and depression to build awareness of affordable and accessible dietary means to combat the mental condition. They obtained a negative association between these micronutrients and depression and emphasized building collaboration between physicians and nutritionists while maintaining caution about the quantity and duration of intake (Zhang et al.).

Another essential micronutrient is zinc, which is required for the nucleic acid metabolism and stability for protein synthesis, gene expression, cell division, and enzyme activity. An imbalance in the diet may lead to mild-to-moderate zinc deficiency (15). Qorbani et al. reported an association between the serum zinc level and low HDL and fasting blood glucose levels but found no significant association between the serum zinc level and metabolic syndrome among children and adolescents in Iran, which may possibly be attributed to race and age, thus warranting future studies on the topic.

Several research works on possible determinants and preventive measures for undernutrition have been included in this “Research Topic.” Ntambara et al. observed that poor nutritional outcomes in children might be prevented with optimal birth spacing, an observation that governments can utilize in policymaking for maternal and child health programs. Zemene et al. noted a relationship of changes in thinness in adolescent girls with age, place of residence, marital status, and toilet facilities. Inadequate feeding may lead to malnutrition among infants under 6 months of age (16). Gessese et al. observed low breastfeeding performance among women experiencing higher socioeconomic conditions and with higher education levels in Ethiopia, meaning that intervention may be needed to improve this breastfeeding performance.

Overnutrition and obesity have also been showing a rising trend in LMICs in recent times (3), and studies need to be carried out to understand the gravity of the situation. Several articles on studies carried out on overweight and obese populations have been included here. Bhattarai et al. noted that in rural Nepal, the overweight and obese population are educated and belong to a high socioeconomic background. A Westernized dietary pattern was reported to be associated with obesity among Mexican men by Rodríguez-Ramírez et al., who have brought to attention the changing trends of diet that are becoming more energy dense and rich in sugar and fat and called for close nutritional monitoring policies. While analyzing a population survey in China from 2014 to 2020, Jiang et al. observed a gradual rise in BMI among elderly men and women, with a significant increase in the obese and overweight elderly population. Chronic diseases, poor dietary choices, lack of exercise, and day-to-day stress may have contributed to the trend (Jiang et al.). Inflammatory markers and metabolic markers such as C-reactive protein, macrophage inflammatory protein-1, and leptin levels were noted to be associated with red and processed meat consumption in obese and overweight Iranian women by Shiraseb et al. The environment of inflammation in the body may eventually lead to various inflammation-related diseases (17).

The resting metabolic rate (RMR) is the most significant component of total daily energy expenditure, which may be altered by ultra-processed food. Ultra-processed food (UPF) may cause a rise in inflammatory markers and alter the RMR. In their study, Bahrampour et al. noted an increase in inflammatory markers such as interleukin-1 beta (IL-1β) monocyte chemoattractant protein (MCP-1), plasminogen activator-1 (PAI-1), and C-reactive protein (hs-CRP) in individuals consuming UPF. Supplementary Table 1 depicts the studies included in the “Research Topic” regarding the determinants of malnutrition (both obesity and undernutrition) and obesity-determining tools. Inflammatory changes disrupt the body's homeostasis and may pave the way for the development of non-communicable diseases (18).

Malnutrition may eventually lead to several non-communicable diseases (NCD), including hypertension, cardiometabolic disorders, diabetes mellitus, dementia, and even cancers (19–22). The nutritional imbalance may trigger insulin resistance, oxidative stress, and inflammation, eventually paving the way for NCD development (23). Nutritional status in early life may be related to cardiovascular disease (CVD) development (24). Keeping this in mind, Yan et al. researched the link between exposure at the early stages of life to the Great Famine in China and hypertrophy of the left ventricle. They observed that the possible mechanisms that may connect undernutrition in fetal life and childhood to this cardiac disorder are catch-up growth once famine is overcome, leading to obesity, endothelial dysfunction, insulin resistance, inflammation, and oxidative stress (Yan et al.).

Consumption of processed food has emerged as a cause of the development of cardiovascular disease (25). Ultra-processed food (UPF) contains components high in total fat, saturated fat, sugar, salt, and energy density and low in vitamins and fiber (26). Dietary, anthropometric, and biochemical assessments were performed by Hosseininasab et al. to observe the relationship between UPF intake and cardiometabolic disorders among obese and overweight women. However, as it was a cross-sectional study, recall bias of the subjects may have presented a limitation (Hosseininasab et al.).

The development of obesity and cardiovascular disease in LMICs may be attributed to insufficient dietary intake, lack of physical activity, urbanization, and socioeconomic factors (27, 28). Food globalization and rapid urbanization, with the dramatic change in dietary patterns to nutrition-poor, processed, energy-dense food, is causing a dramatic rise in dyslipidemia and CVD (29–31). With growing urbanization, the sale of unhealthy street food and poor dietary choices have risen among city dwellers looking for quick meals, which are unfortunately energy dense and high in sugar, sodium, and fats, leading to people's health spiraling down (32). The findings of the research performed by Sousa et al. on the pattern of street food sold by vendors in some bustling cities of Central Asia further re-enforce this understanding. They observed that the food in demand in the city centers is energy dense, while the food in demand in the peripheries is rich in trans and saturated fat. The researchers suggested such findings need to be used for public health interventions (Sousa et al.).

Xiang et al. extracted the sex–age-specific burdens as a result of risk related to the Chinese pattern of food consumption from the “Global Burden of Disease (GBD) Study 2019,” which included “annual and age-standardized rates (ASRs) of death, disability-adjusted life years (DAILYs) and summary exposure values (SEVs) during 1990-2019”.They noted a significant rise in the DALY number and dietary risk-based deaths over the years as the dietary pattern changed. The main reasons for death due to diet were ischemic heart disease, stroke, and colorectal cancers (Xiang et al.).

Vargas-Rosvik et al. assessed the association of dietary intake and sociodemographic factors with adiposity and cardiometabolic disorder among children between 6 and 8 years of age from the urban and rural regions of Ecuador. They highlighted the alarming rise in the risk of cardiometabolic disorder among children (Vargas-Rosvik et al.). Nawsherwan et al. reported the changing pattern and forecasted the burden of cardiovascular disease in the future in East Asia due to risk factors pertaining to diet. They highlighted that a diet high in sodium and low in whole grains and legumes were the major factors contributing to mortality due to ischemic heart disease (Nawsherwan et al.).

In LMICs, studies pertaining to modifiable risk factors, such as sarcopenia, contributing to the development of dementia and its relationship with obesity are few. It is important to bring such risk factors to attention, since improving physical activity and adopting a healthy diet may help prevent such conditions. O'Donovan et al. researched the existing relationship between sarcopenia, obesity, and dementia and observed an association between sarcopenia and dementia; they stressed on the need to develop of health policies that promote a healthy lifestyle. The occurrence of one of the most common malignancies, colorectal carcinoma, is increasingly likely due to the rising consumption of a diet rich in fat, sugar, and food from animal sources (33). Considering this, Seyyedsalehi et al. performed a study to observe the association between dietary fat consumption and colorectal carcinoma. Upon finding an association, they advised the promotion of a decrease in the high intake of fatty acids from industrial and animal sources (Seyyedsalehi et al.). Supplementary Table 2 shows the studies performed to find the link between dietary imbalance and the development of NCD.

Adopting a healthy dietary pattern may aid in lowering the development of non-communicable diseases (34). Several studies related to the influence of the pattern of food consumption on human physiology were included in this “Research Topic”. Branched dietary branched-chain amino acids (BCAA) such as leucine, isoleucine, and valine are significant for protein synthesis, metabolism of glucose, and signaling pathways sensitive to nutrients (35). One study by Zheng et al. researched the association between dietary BCAA risk of cardiovascular diseases (CVD) in individuals with diabetes mellitus in China. They suggested that plant-based BCAA intake can reduce oxidative stress and degradation of protein and lower the risk of CVD (Zheng et al.). Another study was performed using cohort data from the China Health and Nutrition Survey 1997–2009 by Ren et al. to determine whether there is a link connecting intake of BCAA and hyperuricemia risk; they observed an inverse relationship between the two parameters. They noted that over the years, as the dietary pattern has changed, BCAA consumption has reduced, and the risk of hyperuricemia has increased (Ren et al.). Chalermsri et al. assessed the dietary diversity score (DDS) to find a relationship between dietary diversity and the risk of CVD and cardiometabolic disorders among the older population. Their research findings led them to advise the encouragement of a diverse diet for the aging population and the providing of lifelong education promoting a healthy diet to lower the risk of this most common non-communicable disease (Chalermsri et al.). Hypertension is a major risk factor for cardiovascular disease development; thus, it is necessary to take preventive measures to halt its development and progression (36). Diet may play a significant role in this aspect (37–39). Wang et al. thus performed a cross-sectional study, using “the subset of the Suzhou Food Consumption and Health State Survey in 2018–2019”, on the effect of dietary patterns on hypertension. They emphasized that the dietary pattern has a preventive role in the development of hypertension (Wang et al.).

The relationship of dietary insulin load and dietary insulin index with the hypertriglyceridemic waist phenotype and brain-derived neurotrophic factors in Iranian adults was investigated by Hajhashemy et al., who mentioned that even though dietary insulin load and dietary insulin index cannot distinguish between healthy and unhealthy food, dietary insulin load may help lower the hypertriglyceridemic waist phenotype. The risk of diabetes is closely linked to diet; thus, studies need to be performed to learn more regarding the dietary components that may help maintain blood glucose levels. In this regard, Yang et al. conducted a prospective cohort study using the China Health and Nutrition Survey to find a relationship between the consumption of dairy products and the risk of diabetes mellitus. Their finding builds optimism toward a diet containing dairy products that may reduce the risk of diabetes mellitus. Further studies need to be performed, as per the researchers' recommendation, to know the optimal level of dairy consumption to prevent diabetes mellitus (Yang et al.).

It is also important to find certain nutritional supplements to improve the general population's health, emphasizing age groups more susceptible to nutritional imbalance and the development of non-communicable diseases. Two such articles have been included in this “Research Topic”. Adolescence is when nutritional requirements are high, but in LMICs, the poverty-stricken population may find difficulty accessing adequate food with good nutritional value (40). Thus, finding cost-effective dietary supplements may aid in fulfilling their nutritional needs. Khanam et al. studied the effects of Moringa oleifera on hemoglobin, serum retinol levels, and underweight status among adolescent girls in Bangladesh. Moringa oleifera is cost-effective and high in nutritional value, and Khanam et al. suggested that Moringa oleifera should be promoted as part of a regular diet.

Another population susceptible to malnutrition is the elderly population. They often become frail and suffer from different NCDs. Gao et al. performed a 6-year longitudinal study on the effects of tea drinking on frailty among the older Chinese population. Tea drinking is an integral part of Chinese culture, and a negative correlation was found between tea drinking and the development of frailty. Their findings should be considered, and tea-drinking habits should be encouraged among the older population of LMICs (Gao et al.). Supplementary Table 3 shows the studies on dietary patterns that may positively impact human health.

The various research materials included in this “Research Topic” bring to light the recent changes in dietary patterns and their alarming consequences on the population of LMICs. The boost in urbanization has paved the way for demands for fast food high in energy, fat, and sugar. This is causing a significant portion of the population to become obese and develop life-altering NCDs, which is creating a personal, social, and government burden. On the other hand, some of the population suffers from undernutrition due to poverty and a lack of proper understanding. Several studies have repeatedly stressed the need for a healthy balanced diet and should be given importance. The LMICs need to develop policies to combat this emerging danger of malnutrition to secure a healthy and stable future for their population.

## Author contributions

All authors listed have made a substantial, direct, and intellectual contribution to the work and approved it for publication.
