# Supplementary material for: Editorial: Diet and nutrition for non-communicable diseases in low and middle-income countries
Source: Front Nutr. 2023 Mar 28;10:1179640. doi: 10.3389/fnut.2023.1179640 (PMC10088507; doi:10.3389/fnut.2023.1179640)
Supplement: Supplementary file 1 [file Table_1.docx]

| Reference | Study Population | Study Design | Study Period | Subgroup | Results |
| --- | --- | --- | --- | --- | --- |
| Oumer et al 2022 | N=380 pregnant women  Mean age=28 years | health facility-based survey | 1 month | Study identified three dietary patterns.  1.animal-source foods and  Fruits  2.cereals, tubers, and sweety foods  3.pulses (legumes)  and vegetables” | Women with absence of food aversion (AOR = 1.59; 1.08–2.35), snacks (AOR = 1.93; 1.23–2.75), non-fasting (AOR = 0.75; 1.12–2.12), and receiving nutritional counseling (AOR = 1.96; 1.25–3.07) were significantly associated with a greater intake of fruits and animal-source food consumption. Aversion (AOR = 1.60;1.04–2.44) , Non-working mothers (AOR = 1.8;1.23–2.76), chronic disease (AOR = 1.88; 1.14–3.09), were fasting (AOR = 1.33;0.88–2.01), nor received nutritional counseling (AOR = 1.33; 0.88–2.01), and absence of food  cravings (AOR = 4.27;2.67–6.84) had significantly greater chances of consuming cereals, tubers, and sweet foods. Greater odds of legume and vegetable consumption was found among those with Literacy (AOR = 1.87; 1.14–3.09), low socioeconomic class (AOR = 2.68;1.30–5.23), skipping meals (AOR = 1.73; 1.15–2.62) and urban residence (AOR = 2.10; 1.10–3.93) |
| Al-Jawaldeh et al. 2022 | N=19 EMR member countries | situational analysis of the regulatory framework of food marketing policies targeting  children in the Eastern Mediterranean Region (EMR). An overview of the legislation and Implementation of restrictions relevant to the WHO Recommendations. | 2005-2021  (16 years) |  | Legislation for media outlets was 14%, 41% of countries had restrictions in nurseries and schools’ canteens . No country implemented a comprehensive regulatory approach to limit marketing of unhealthy food and beverages to children observed using the mapping exercise .  Iran, KSA, Oman, Pakistan, Qatar, Tunisia, and the UAE have begun planning and/or implementing some levels of reforms. Iran adopted most reforms compared to the remaining other counties. |
| Hu et al 2022 | N= 36,950 adolescents  Mean age= 10.23 ± 2.77 | Retrospective Cohort study | 1 year  [2020] | TMI cutoffs to define,  adolescent overweight and obesity were 13.1 and 14.1 kg/m3  for participants under 16, respectively. The corresponding  TMI cutoffs for those aged 16 or over were 14.0 and 15.8 kg/m3,  respectively. | TMI had predictive abilities that were better than BMI among all of the  participants for hypertension prediction (difference in AUC = 0.019, 95% CI = 0.007–0.031; NRI = 0.067, 95% CI = 0.008–0.127) and isolated systolic hypertension (difference in AUC = 0.021, 95% CI = 0.005–0.036; NRI = 0.106, 95% CI =  0.029–0.183). The difference in abilities of prediction between BMI and TMI was more apparent in the ≥16 years age group. Also, TMI performed better than BMI in prediction of adolescent hypertension in girls but not in boys. |
| Zhang et al. 2022 | N= 25 observational studies (100,955 participants), which included  24 cross-sectional/case-control and 1 prospective cohort study | meta-analysis of observational studies on the associations of  dietary vitamin A and beta-carotene intake with depression. | studies published between 2009.  and 2022. |  | Intake of vitamin A and beta carotene was inversely related to depression (RR = 0.83, 95%CI: 0.70–1.00;P = 0.05). Combined SMD exhibited the dietary vitamin A intake in depression was less than that of control (SMD = −0.13, 95%CI:−0.18 to −0.07; P < 0.001). The multi-variable adjusted RR  showed that dietary beta-carotene intake was negatively related to depression(RR = 0.63, 95%CI: 0.55–0.72; P < 0.001). The combined SMD showed that the dietary beta-carotene intake in depression was less than that in control (SMD = −0.34, 95%CI: −0.48 to −0.20; P < 0.001). |
| Qorbani et al 2022 | n= 1371;  mean age= 12.24 ± 3.23 yrs | Cross sectional study | 12 years  1997–2008 | Statin  Case:2,388 | 12.40% (n = 170) suffered metabolic syndrome, of which 55.7% were boys and 44.3% were girls.  Mean zinc levels (µg/dL) in patients with and without metabolic syndrome  were 107.03 and 110.6, respectively (p-value = 0.211) and 111.8 for boys and  109.10 for girls (p-value = 0.677). |
| Ntambara et al. 2022 | N=898,860  Age= 36-48 months | Meta-analysis of 48 studies | 1998-2022 |  | Compared with a short birth interval of <24 months, birth interval of ≥24 months and risk of being underweight showed a U-shape that the optimum birth interval group of 36–48 months yielded the most protective efect (OR = 0.54, 95% CI = 0.32–0.89).Birth interval of ≥24 months was significantly associated with decreased risk of stunting (OR = 0.61, 95% CI = 0.55–0.67) and wasting (OR = 0.63, 95%CI =0.50–0.79) when compared with the birth interval of <24 months. |
| Zemene et al. 2022 | N=11783  Age= 15-19 years | Secondary data analysis was done based on the  four consecutive Ethiopian Demographic and Health Survey  (EDHS) datasets | 16 years  2000-2016 |  | Thinness among late adolescent girls significantly reduced from  34.4% (95% CI: 32.8%, 36.0%) in 2000 to 24.9% (95% CI: 23.4%, 26.5%) in 2016 with an annual average decline rate of 1.73%. About 84% of the decline in thinness was attributed to the change in the effect of the characteristics.  The place of residence and marital status were significantly associated with a change in thinness due to the change in coefficients. The compositional changes in the age of the adolescents, religion, and types of toilet facilities were also significantly associated with the change in thinness. From the  multilevel binary logistic regression, higher age of adolescents (AOR = 0.83;  95% CI: 0.77, 0.90), improved toilet facility (AOR = 0.45; 95% CI: 0.31, 0.65),middle wealth index (AOR = 1.45; 95% CI: 1.10, 1.90), and female head of the household (AOR = 0.77; 95% CI: 0.61, 0.98) were significantly associated at an individual level, whereas being from Somali (AOR = 2.14; 95% CI:1.76, 3.10) |
| Gessese et al. 2022 | N= 4,273;  mean age= 45.8 years | Cross sectional Study using  2019 Ethiopia  Mini Demographic and Health Survey (EMDHS) dataset. | 1 year  (2019) |  | Low breastfeeding performance index was 79.05% (95% CI: 78.01, 81.59). A unit increase in child age (AOR = 11.56; 95% CI: 6.97, 19.17), the richest wealth quintile (AOR = 2.76; 95% CI: 1.18, 6.5), a higher level of education(AOR = 5.41; 95% CI: 2.08, 14.05), being married or living with partner (AOR= 2.73; 95% CI: 1.18, 6.27), being women from Somali (AOR = 5.11; 95% CI:  2.08, 12.56), Afar (AOR = 3.03; 95% CI: 1.16, 7.91), Oromia (AOR = 1.88; 95%CI: 1.03, 3.41), Diredawa city administration (AOR = 2.89; 95% CI: 1.04, 8.07),and antenatal care (ANC) visit (AOR = 2.05; 95% CI: 1.31, 3.19) were positively associated with the low breastfeeding performance index. |
| Bhattarai et al. 2022 | N= 260  Age= ≥18yrs | Cross sectional study | 3 months  (October to December 2016) |  | The age-standardized prevalence of overweight/obesity was higher  for individuals with higher education (23%) and high-income (32%) and those  who were unemployed (42%). Compared to the low-income and no formal education groups, the prevalence ratio of overweight/ obesity was 1.69 and 2.27 times more for those belonging to the high-income and high school and  above groups, respectively. No evidence of effect modification by gender and age was observed |
| Rodríguez-Ramírez et al 2022 | N= 5,735  Age=20-59 | Cross sectional study | May to October 2016 | Participants were classified as having normal weight, overweight-obesity and  by their abdominal circumference as having abdominal obesity or not. | In men, Westernized pattern was related to overweight-obesity (PR =1.11, 95% CI 0.97–1.27), and abdominal obesity (PR = 1.15, 95% CI 1.00–1.33), the Diverse pattern was associated with overweight-obesity (PR = 1.18, 95% CI 1.00–1.38),  and abdominal obesity (PR = 1.27, 95% CI 1.07–1.50), compared with the Rural pattern.  In women, these dietary patterns were not associated with obesity. |
| Jiang et al. 2022 | N= 50192; Age= 71.9 ± 6.1 yrs | Cross sectional study | 6 years  2014–2020 | underweight (BMI < 18.5 kg/m2), normal weight  (18.5 kg/m2 ≤ BMI < 25 kg/m2), overweight (25 kg/m2 ≤ BMI < 30 kg/m2), and obesity  (BMI ≥ 30 kg/m2). | BMI gradually increased from 2014 to 2020.  The age-adjusted mean BMI increased by 0.3 kg/m2 in older men, and 0.5 kg/m2 in older women. The age- and sex-standardized prevalence of obesity and overweight significantly higher particularly in 70–79-year age group, while the prevalence of underweight lowered. |
| Shiraseb et al. 2022 | N=391 overweight and obese women  Age=18-56 years | Cross sectional study | 1 year  (2018) |  | higher intake of processed meat had a significant positive association with leptin levels (β:0.900, 95% CI: 0.031;1.233, p = 0.015).  Positive significant associations between high-  sensitivity C-reactive protein (hs-CRP) (β:0.020, 95% CI:0.000;0.050, P = 0.014) and plasminogen activator inhibitor 1 (PAI-1) (β:0.263, 95% CI:0.112;0.345, p= 0.053) and MCP-1 (β:0.490, 95% CI: 0.175;1.464, p = 0.071) levels with red meat were also shown; Significant positive association between processed meat and macrophage inflammatory protein (MCP-1) levels was noted (β: 0.304, 95%  CI:0.100;1.596, p = 0.025). There was a significant negative association between red meat and the homeostasis model assessment of insulin resistance(HOMA-IR) (β: −0.016, 95% CI: −0.022, −0.001, p = 0.033).Significant negative association was found with Galectin-3 (Gal-3) (β: −0.110, 95% CI: −0.271;0.000, p =0.044), MCP-1 (β: −1.933, 95% CI: −3.721;0.192, p = 0.022) and Homeostatic Model Assessment for Insulin Resistance (HOMA-IR) (β: −0.011, 95% CI:−0.020,0.000, p = 0.070) levels with high adherence of white meat intake. Significant marginal positive association between PAI-1 levels and high adherence to white meat intake (β: −0.340, 95% CI: −0.751;0.050, p = 0.070) was found |
| Bahrampour et al. 2022 | N= 285;  age= 18-48yrs | Cross sectional study in Tehran, Iran |  |  | A negative association between the Resting Metabolic Rate (RMR) [β = −0.159, 95%  confidence interval (CI): −0.471, −0.052, P = 0.044], RMR per BMI (β = −0.014,95% CI: −0.025, −0.006, P = 0.036), and RMR per Free Fat mass (FFM) (β = −0.241, 95%CI: −0.006, −0.000, P = 0.041) |
