# Supplementary material for: Editorial: Diet and nutrition for non-communicable diseases in low and middle-income countries
Source: Front Nutr. 2023 Mar 28;10:1179640. doi: 10.3389/fnut.2023.1179640 (PMC10088507; doi:10.3389/fnut.2023.1179640)
Supplement: Supplementary file 2 [file Table_2.docx]

| Reference | Study Population | Study Design | Study Period | Subgroup | Results |
| --- | --- | --- | --- | --- | --- |
| Yan et al. 2022 | N= 2,543.  mean age= 59.07 ± 3.65 yrs | Cross sectional study to find association between the exposure to famine and left ventricular hypertrophy | 1 year  (01/01/2017 to 31/12/2018) | The  Participants exposed to the great Chinese Famine were classified into five groups as follows,  (1) non-, (2) fetal-, (3) early-, (4) mid-, and (5) late-childhood  exposed groups, which represents participants born between  01/10/1962 and 30/09/1964 (n = 517), 01/10/1959 and 30/09/1961  (n = 346), 01/10/1956 and 30/09/1958 (n = 488), 01/10/1954  and 30/09/1956 (n = 630), and 01/10/1952 and 30/09/1954  (n = 562), respectively. | LVH prevalence was 122 (23.6%),  87 (25.1%), 133 (27.3%), 184 (29.2%), and 178 (31.7%), in non-, fetal-, early-, mid-, and late-childhood exposed groups, respectively (p = 0.031), while in the non-exposed group, the ORs for developing carotid plaque as a result of fetal, early-, mid- to late-childhood exposure was 1.08 (95% CI: 0.76, 1.59, p = 0.619), 1.24 (95% CI: 1.03, 1.79, p = 0.031), 1.49 (95% CI: 1.10, 2.01, p = 0.009), and 1.64 (95% CI: 1.25, 2.18, p=0.001), respectively (p for trend = 0.003). |
| Hosseininasab et al.2022 | N=391  Age=18-65 | Cross sectional study | 1 year  (2018) |  | Significant association between UPF consumption  and transforming growth factor (TGF) (β: 0.101, 95% CI: 0.023, 0.180, p =  0.012), atherogenic coefficient (AC) (β: 0.011, 95% CI: 0.001, 0.032, p = 0.034),  visceral fat level (VFL) (β: 0.006, 95% CI: −0.017, 0.029, p = 0.076), and the  quantitative insulin sensitivity check index (QUICKI) (β: −3.775, 95%CI: 0.001,  0.001, p = 0.042). |
| Sousa et al. 2022 | N=714 customers who purchased 852 food,  age= 70 yrs | Cross sectional study | 1 year  2016-2017  In cities of Tajikistan, Kyrgyzstan, Turkmenistan and  Kazakhstan respectively Dushanbe (between April and May  2016), Bishkek (June and July 2016), Ashgabat (October 2016)  and Almaty (August 2017). |  | Customers’ influx, buying rate and purchase of industrial food were higher in  city centers compared to the outskirts (median: 4.0 vs. 2.0 customers/10 min, p < 0.001. 5.0 vs. 2.0 food items/10 min, p < 0.001; 36.2 vs. 28.7%, p = 0.004). Tea, coffee, bread and savory pastries were most frequently purchased in the early morning, bread, main dishes and savory pastries during lunchtime, and industrial products in the mid-morning  and mid-afternoon periods. Energy and macronutrient density was highest at 11:00–12:00 and lowest at 09:00–10:00. Purchases were smaller but more energy-dense in city centers, and higher in saturated and trans-fat in the peripheries. |
| Xiang et al. 2022 |  | Systemic analysis using Data on annual numbers and age-standardized rates (ASRs)  of sex-age-specific death and Disability adjusted life years (DALYs) due to dietary risk from  1990 to 2019 | 19 years (1990-2019) |  | Between 1990 and 2019, the dietary risk-based death and  DALYs significantly raised in China with a trend spiraling downward of ASDR  and ASR-DALYs. Ischemic heart disease was found as the first cause of death from diet, followed by stroke and colon and rectum cancers. Greater risk of diet related death and DALY were noted in Chinese men than women.  The number 1 cause for demise among the Chinese population was noted to be diet high in sodium. with increasing age the death burden risk rose which peaked in those above the age of 75 years. Diet similar to the Chinese including high sodium intake was observed among the residents of Japan and South Korea. Lowered whole grain intake ranked second in China’s dietary risks. |
| Vargas-Rosvik et al. 2022 | N=267; Average  age= 6.7 ± 0.7 yrs | Cross sectional study | 6 months  (February- August 2018) | Children living in urban region  Children from rural area in Ecuador | overweight or obese children: 29%, 12% had low HDL levels,  and over 18% had high levels of LDL and triglycerides. Children living in the urban region had lower HDL level(β −4.07 mg/dL; 95% CI: −7.00; −1.15;P = 0.007) but higher LDL cholesterol level (β 8.52 mg/dL; 95% CI: 1.38;  15.66; P = 0.019). Hepatic enzymes were higher among urban children  (SGOT: β% 22.13; 95% CI: 17.33; 26.93; P < 0.001; SGPT: β 0.84 U/L; 95% CI: 0.09; 1.59; P = 0.028). Higher Leptin blood levels (β% 29.27; 95%CI: 3.57; 54.97; P = 0.026),adiponectin plasma concentrations were lower among urban children (β%−103.24; 95% CI: −58.9; −147.58;  P = < 0.001). Fiber intake was inversely associated with total cholesterol (β−9.27 mg/dL; 95% CI –18.09; −0.45; P = 0.040) and LDL cholesterol blood levels (β−9.99 mg/dL; 95% CI: −18.22; −1.75; P = 0.018). |
| Nawsherwan et al. 2022 | N= 100,100.  age: ≥50 yrs | Systemic analysis of The Global Burden of Disease (GBD) 2019 data | 19 years  (1990–2019) | Average Annual Percentage Changes (AAPC)s  for each decade (i.e., 1990–1999, 2000–2009, and 2010–2019).  Based on age groups (i.e., 50–54, 55–59, 60–64, 65–69, 70–74, and 75–79 years), AAPC for IHD and Ischemic Stroke burden was obtained for both males and females from 1990 to 2019. | Age-standardized mortality rate (ASMR) and Disability adjusted life years (DALYs) of Ischemic heart disease (IHD)attributed to dietary risk factors significantly decreased in Japan, South Korea, and the world by 3.4% (95% CI: −3.6,−3.3), 4.9% (95% CI: −5.2, −4.7) and 1.4% (95% CI: −1.6,  −1.3) per year. ASMR of IHD significantly rose in Chinese males and for both sexes in North Korea 0.4% (95% CI: 0.2,0.6) and 0.5% (95% CI: 0.3, 0.6) per year. ASMR and DALYs of IHD due to dietary risk factors were more in males than in females globally. From 2020 to 2030, the ASMR of IHD is expected to rise in South Korean females and Japanese males. A diet low in whole grains was the number 1 risk factor for the highest IHD mortality and DALYs in 2019, followed by a diet low in legumes and a diet high in sodium. The leading risk factors for high IHD mortality in East Asian countries were diet low in whole grains, a diet low in legumes and a diet high in sodium were |
| O’Donovan et al. 2022 | N=5,760  Mean age= 71 ± 8 years | Cross sectional study. Data were from the National Survey of  Health, Wellbeing and Aging in Colombia | 6 months  (April-September 2015) | Overweight and Obese  Normal BMI  Underweight | Mild cognitive impairment prevalence 17% in 5,760 participants  in which sarcopenia was assessed. Overweight and obesity were associated with lowered risk of mild cognitive impairment and increased risk was associated with sarcopenia. Sarcopenia was a risk factor for mild-cognitive impairment in those with normal BMI. Compared with those with normal BMI and without sarcopenia, the odds ratio was 1.62 in those with obesity and sarcopenia (95% confidence interval: 1.07, 2.48). Compared with those with normal BMI and without sarcopenia, the odds ratio for mild cognitive impairment was 1.84 in those with normal BMI and sarcopenia (95% confidence interval: 1.25, 2.71). Sarcopenia was also a risk factor in those with obesity but did not present a greater risk than sarcopenia alone. Sarcopenia was not a risk factor for mild cognitive impairment in those with overweight. |
| Seyyedsalehi et al. 2022 | N=4071  Mean age= 58.5 years (case)  57.1 years(control) | case-control study | 3 years  (May 2017 and 2020) | 865 cases  3206 controls | positive association was noted between Colorectal Cancer (CRC) and high intake of dietary total fat (OR highest quartile Q4 = 1.77, 95% CI = 1.32–2.38), palmitoleic acid (ORQ4 = 2.16, 95% CI= 1.19, 3.91), and cholesterol(ORQ4 = 1.58, 95% CI = 1.22–2.05) and an inverse association with high intake of dietary heptanoic acid (ORQ4 = 0.33, 95% CI = 0.14, 0.79) and low intake of palmitic acid (OR lowest quartile Q2 = 0.53, 95% CI = 0.31–0.88). |
