# Supplementary material for: Editorial: Diet and nutrition for non-communicable diseases in low and middle-income countries
Source: Front Nutr. 2023 Mar 28;10:1179640. doi: 10.3389/fnut.2023.1179640 (PMC10088507; doi:10.3389/fnut.2023.1179640)
Supplement: Supplementary file 3 [file Table_3.docx]

| Reference | Study Population | Study Design | Study Period | Subgroup | Results |
| --- | --- | --- | --- | --- | --- |
| Zheng et al. 2022 | N= 838 patients with type 2 diabetes who have  been diagnosed with CVD (within 2 weeks)  Mean age=62.08 years | case–control  study | 3 years  (2013-2015) | Case=419  Control=419 | Dietary BCAA was inversely associated with Cardiovascular disease(CVD) risk (OR Q4−Q1 = 0.23, 95%CI =0.10, 0.51, P value= <0.001 for total BCAA; OR Q4−Q1 = 0.20, 95%CI = 0.07,0.53, P value = 0.001 for leucine). For each 1-S.D. increase in total dietary BCAA, leucine or valine intake was associated with 54% (95%CI = 29%, 70%, P=0.001), 64% (95%CI = 29%, 82%, P=0.003), or 54% (95%CI = 1%, 79%, p=0.049) decrease in the risk of CVD, respectively. Whole grains, starchy |
| Ren et al. 2022 | N=6810.  mean age= 42.5 ± 13.4 yrs | Cohort study | 12 years  (1997–2009) | Participants were designated into four subgroups on basis of the trajectories of dietary BCAA consumption.  T1: Low stable  T2: High to low  T3: Moderate stable  T4: Moderate to high then  Decline | Compared with the low stable trajectory group, high to low trajectory group was greatly related to an increased risk of Hyperuricemia (HU) [HR 1.35 (95% CI 1.03 to 1.79)] with modification for covariates. Total cholesterol (TC), hemoglobin A1c (HbA1c), fasting blood glucose (FBG), and triglyceride (TG) partially regulated trajectories and HU. |
| Chalermsri et al. 2022 | N=6,956  Mean age=68 years | cross-sectional study using the sub-sample of the fifth Thai National Health Examination Survey | 3 years  (2013-2015) |  | Dietary Diversity score (DDS) had a significant negative association with  log-Thai Cardiovascular risk score, with adjusted β (95% CI) values of −0.01 (−0.01, −0.01).Regarding the cardiometabolic risk factors, DDS had a significant negative association with hypertension, Diabetes Mellitus(DM) and log-TG levels, with adjusted OR (95%CI) values of 0.97 (95% CI 0.97, 0.98) for hypertension, 0.94 (0.93, 0.95) for DM, and adjusted β (95% CI) values of −0.002 (−0.004, −0.001) for log-TG level.  DDS was positively associated with TC and LDL-C, with adjusted β (95% CI)  values of 0.59 (0.38, 0.80) for TC and 0.59 (0.38, 0.79) for LDL-C levels, while  DDS was not associated with HDL-C level. |
| Wang et al. 2022 | N=2718.  age= ≥18 yrs | Cross sectional study in Eastern China | 1 year (2018-2019) |  | fruit-dairy pattern was inversely associated with hypertension  after adjustment for all the covariates (OR = 0.55; 95% CI: 0.40, 0.75; P = 0.002) |
| Hajhashemy et al 2022 | N=528.  age= 42.5 (±11.1) yrs | Cross sectional study | 1 year  (2021) |  | Individuals in the top tertile of Dietary Insulin Load(DIL), in comparison to those in the bottom tertile, had higher odds of hypertriglyceridemic waist phenotype (HTGW) (OR = 6.10, 95% CI: 1.58–23.53)  After considering confounders, participants  in the top tertile of DIL had slightly more odds of having low brain-derived neurotrophic factor (BDNF) values (OR = 2.00, 95% CI: 0.95–4.21). Association between DII and odds of low BDNF values was statistically insignificant. |
| Yang et al. 2022 | N= 15,512.  age= ≥ 18 years | Secondary analysis of prospective cohort study | 18 years (1997 to 2015) |  | Participants who had no dairy consumption were 12,368 (79.7%),  while 2,179 (14.0%) and 947 (6.1%) consumed dairy at 0.1–100 and >100 g/day, respectively. Dairy consumption of  0.1–100 g/day was associated with lower risk of diabetes in all participants (HR 0.53, 95% CI:0.38 −0.74; P < 0.001) and males (HR 0.50, 95% CI: 0.31–0.80; P=0.004). Significant protective effect on diabetes in the total population with dairy consumption ranged from 25 to 65 g/day (HR < 1, P = 0.025). Intake of 30–80 g/day was related to lowered diabetes risk among the ≤2,000 kcal/day energy intake group (HR < 1, P = 0.023). Inverse association was found between dairy consumption and  risk of diabetes in Chinese population |
| Khanam et al. 2022 | N= 226.  Mean age of interventional group= 12.7 ± 0.7yrs  Mean age of control group=13.3 ± 0.8 yrs | Quasi Experimental study | 1 year | Interventional group=113  Control group=113 | Upon intervention with Moringa Oleifera significant positive changes in Hemoglobin (intervention vs. Control: coef = 0.41, P = 0.010) and serum retinol (coef = 0.27, P=0.00). No significant changes in weight were observed between groups |
| Gao et al. 2022 | N=2473;  mean age= 76.88 yrs | Analysis of longitudinal data from 2008 to 2014  of the Chinese Longitudinal Healthy Longevity Survey (CLHLS) | 6 years  (2008-2014) | Non tea drinkers :35.8%  Inconsistent tea drinkers:38.8% Consistent tea drinkers:11.2%  Consistent daily tea drinkers: 14.1% | Compared to non-tea drinkers,  consistent daily tea drinkers reported a significantly lower ratio of having frailty [risk ratio (RR) = 0.54, 95% confidence interval (CI): 0.38–0.78],  consistent daily tea  consumption significantly reduced the risk of frailty for males (RR = 0.53,95% CI: 0.32–0.87) but not females (RR = 0.65, 95% CI: 0.37–1.12); in the young (RR = 0.40, 95% CI: 0.22–0.74) but not in the oldest (aged ≥ 80) (RR= 0.66, 95% CI: 0.40–1.06); informal education (RR = 0.48, 95% CI: 0.28–  0.84) but not formal education (RR = 0.62, 95% CI: 0.37–1.03); financial dependence (RR = 0.42, 95% CI: 0.25–0.71) but not financial independence  (RR = 0.71, 95% CI: 0.41–1.23). Additionally, females showed a lower tea-  mediated risk of frailty in occasional tea consumers (RR = 0.51, 95% CI:0.29–0.89) and inconsistent tea drinkers (RR = 0.58, 95% CI: 0.37–0.93). |
